# Stress inhibits tryptophan hydroxylase expression in a rat model of depression

**DOI:** 10.18632/oncotarget.18780

**Published:** 2017-06-28

**Authors:** Yi Chen, Haixia Xu, Mingyue Zhu, Kun Liu, Bo Lin, Ruxian Luo, Chuanbai Chen, Mengsen Li

**Affiliations:** ^1^ Key Laboratory of Molecular Biology, Hainan Medical College, Haikou 571199, Hainan Province, P. R. China; ^2^ Hainan Provincial Key Laboratory of Carcinogenesis and Intervention, Hainan Medical College, Haikou 571199, Hainan Province, P. R. China; ^3^ Department of Psychiatry, Hainan Provincial Anning Hospital, Haikou 571199, Hainan Province, P. R. China

**Keywords:** depressive model, tryptophan hydroxylase, epigenetics

## Abstract

Serotonin (5-hydroxytryptamine, 5-HT) dysfunction is associated with the pathophysiology of depression. Tryptophan hydroxylase (TPH), the rate-limiting enzyme in 5-HT biosynthesis, is believed to have essential role in many mental disorders, including depression. In the present study, we generated a rat model of depression by exposing the animals to stress, and the rats were then treated with paroxetine. The results indicated that the concentration of 5-HT in the brain and liver tissues were significantly lower in the rat model of depression than in healthy or treated rats. Immunohistochemical analyses of TPH1/2 showed less TPH1 and TPH2 expression, specifically TPH2, in the brain, liver and kidney of the depressive rats than in the healthy rats; In addition, the two TPH isoforms, TPH1 and TPH2, had different spatial distributions,the mRNAs of the TPH1/2 genes were significantly decreased and TPH1/2 were highly methylated in the depressive model rat, but treatment with paroxetine ameliorated the expression and methylation of TPH1/2. All together, stress was able to inhibit expression of TPH1/2 in brain tissue and decrease concentration of 5-HT, the mechanism maybe involve in increasing the methylation of *TPH2* genes promoter; Paroxetine has a role in confronting the effect of stress in depressive rat model.

## INTRODUCTION

Depression is a heritable neuropsychiatric syndrome with a high mortality. Although it is a severe and recurrent psychiatric disorder that affects approximately 10% of people during their lifetime [1, 2], the aetiology of depression is still undefined. The current concept of psychiatric disorders posits that both an individual physiological characteristics (e.g., neurotransmitters and neuroendocrine alterations, and genetics) and environmental factors (e.g., society and family) contribute to depression. Although most serotonin (5-HT), one of the best-known neurotransmitters, is distributed peripherally, and 5-HT levels and signalling have been shown to regulate bone mass in mice and humans [3], 2% of this neurotransmitter is present in the central nervous system and is associated with temperature regulation, circadian rhythmicity, vomiting, aggression, and energy balance [4, 5]. Dysfunction in 5-HT is associated with the pathophysiology of depression [6–10], and the 5-HT system is a common target of antidepressants.

Biosynthesis of 5-HT is a two-step process; the first and rate-limiting step is conversion of the amino acid L-tryptophan into 5-hydroxytryptophan (5-HTP), which is catalyzed by tryptophan hydroxylase (TPH). Two TPH isoforms (TPH1 and TPH2) have been identified and share 71% homology in their protein sequence; The protein sequences of TPH1 and TPH2 differ at their N-terminal [11] and exhibit different spatial distribution patterns [12]. TPH1 is primarily located in a variety of non-neuronal cells, such as the enterochromaffin cells of the gut and the pineal gland [13–16], whereas TPH2 is predominantly expressed in the myenteric plexus [17] and in the serotonergic neurons of the raphe nuclei. TPH acts as the rate-limiting enzyme in 5-HT synthesis [14–16, 18–20]. No substantial overlap has been reported between the expression of the TPH1 and TPH2 proteins in adults [20, 21], supporting the hypothesis that TPH1 primarily catalyzes the synthesis of peripheral 5-HT, whereas TPH2 functions primarily at central serotonergic neurons.

The location and function of TPH2 has made it an essential candidate gene in many mental disorders, including depression. Currently, the majority of research on TPH2 has focused on gene polymorphisms. However, a few studies have examined which TPH2 gene variant is tightly associated with the aetiology of depression [22]. According to several researchers, epigenetic modification of the *TPH2* gene may contribute to modulation in the expression of TPH2 in central neurons, but confirmatory evidence is lacking. In the present study, we hypothesize that TPH2 regulates the expression of 5-HT by epigenetically modulating the *TPH2* gene. In the process of the metabolism and excretion of antidepressive drug, paroxetine and 5-HT are executed by liver and kidney, and the expression of TPH1/2 in these tissues influence 5-HT level. We examined the levels of 5-HT, the expression of TPH protein and mRNA, their immunohistochemical localization, and TPH methylation to assess this hypothesis and determine whether 1) TPH1/2 expression was associated with depression and 2) the level of the TPH1/2 proteins showed the same trend as that of mRNAs transcription from the *TPH1/2* gene.

## RESULTS

### Stress inhibited 5-HT production in various rat tissues

**Figure 1:**
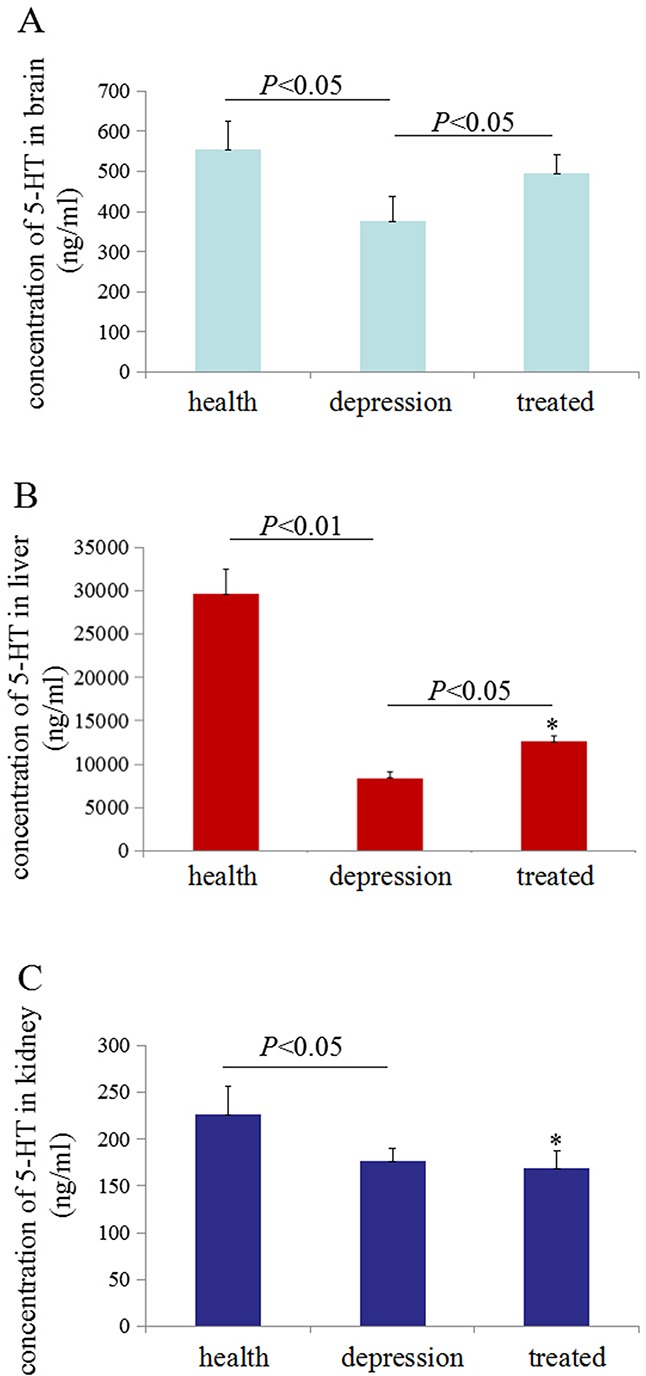
Serotonin (5-HT) concentrations in rat tissues Enzyme-linked immunosorbent assays (ELISAs) were used to detect 5-HT concentration in rat tissues. The 5-HT concentrations were measured in 100 μg of total proteins extracted from the brain **(A)**, liver **(B)** and kidney **(C)** of the rats. *P<0.05 vs healthy group. N=6.

A comparison of 5-HT concentrations in healthy, depressive, and treated rats was performed using ELISAs. Less 5-HT was observed in brain and liver of the depressive model rat than the healthy and treated rats, but the 5-HT concentrations were significantly increased (*P*<0.05 compared with the depressive model group) following treatment with paroxetine (Figure 1A, 1B). Additionally, stress may also inhibit production of 5-HT in the kidney of depressive model rats (Figure 1C). These results for the 5-HT level in our experiments were consistent with the neurotransmitter theory of depression, which proposes that 5-HT synthesis is decreased in the brains of patients with depression [23–26]. However, paroxetine treatment could partially restored 5-HT production in the brain and liver tissues.

### Stress inhibited TPH1/2 expression in various rat tissues

**Figure 2:**
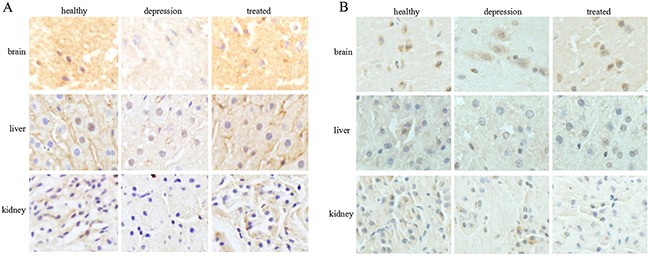
Expression and localization of TPH1/2 in rat tissues The expression and localization of TPH1 **(A)** and TPH2 **(B)** were analyzed by immunohistochemistry in the rat brain, liver, and kidney of the healthy, depressive model, and treated groups. The images are representative of six independent experiments.

**Figure 3:**
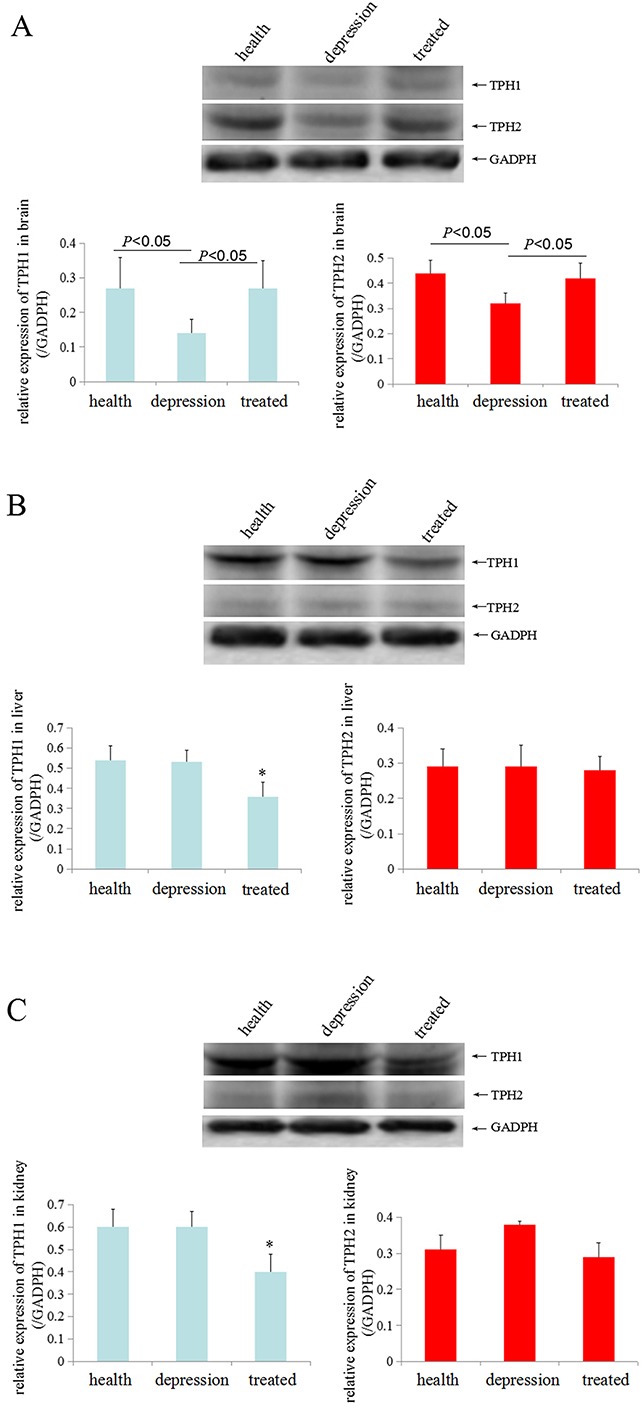
TPH1/2 expression in rat tissues TPH1/2 expression in the brain **(A)**, liver **(B)** and kidney tissues **(C)** from the healthy, depressive model and treated rats was detected by Western blotting. GAPDH (glyceraldehyde-3-phosphate dehydrogenase, a housekeeping gene that is stably expressed in all tissues) was used as an internal control. The images are representative of six independent experiments. *P<0.05 vs healthy and depressive model groups.

To investigate the expression of TPH in distinct tissues of the healthy, depressive, and treated rats, immunohistochemistry and Western blot analyses were performed sequentially. Immunohistochemistry results indicated that the expression of TPH1 was significantly lower in brain, liver and kidney tissues of the depressive model rat than in the healthy and treated rats (Figure 2A). The expression of TPH2 was significantly lower in the brain tissues of the depressive model rat than in the healthy and treated rats, but fewer changes were observed in the liver and kidney tissues from these groups (Figure 2B). Based on the Western blotting results, TPH1 and TPH2 were expressed at significantly lower level in the brain tissues of the depressive model group than in the healthy or treated rats (Figure 3A). Unexpectedly, TPH2 expression showed little change in the liver and kidney tissues of these groups, but TPH1 expression was not changed in the depressive model group compared to the healthy group. However, TPH1 was expressed at significantly lower levels in the liver and kidney tissues from the treated group compared to the healthy and depressive model groups (Figure 3B, 3C). Thus, stress principally inhibited 5-HT biosynthesis by suppressing TPH2 expression in the brain.

### Stress inhibited the expression of TPH1/ 2mRNAs in rat brain tissues

**Figure 4:**
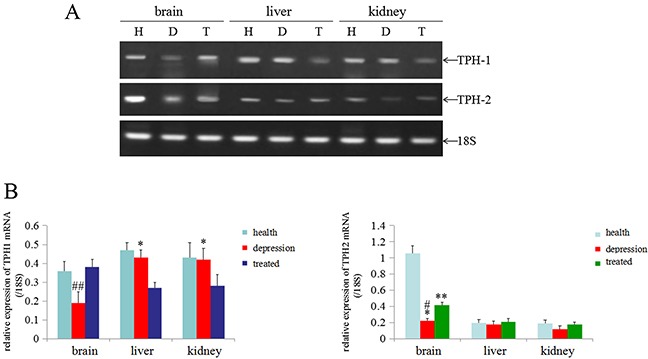
Expression of the TPH1/2 mRNAs in rat tissues **(A)** RT-PCR analyses were used to measure the expression of the TPH1/2 mRNAs in the brain, liver and kidney of healthy, depressive modeland treated rats; 18S was used as an internal control. **(B)** The relative quantity of TPH1/2 mRNAs from RT-PCR (from the images shown in A), ^##^*P*<0.01 vs healthy and treated groups, *P<0.05 vs treated group, **P<0.01 vs healthy group, ^#^*P*<0.01 vs healthy group. H: Healthy rats; D: depressive model rats; T: depressive model rats treated with paroxetine. The images are representative of six independent experiments.

In the present study, we used RT-PCR analyses to detect the levels of the TPH1/2 mRNAs in the brain, liver and kidney tissues of the experimental rats and to explore the effects of stress on TPH1/2 expression. According to the RT-PCR results, the levels of both the TPH1 and TPH2 mRNAs were significantly lower in the brain tissues of the depressive model group than in the healthy or treated group, and the levels of TPH1 mRNA in the liver and kidney tissues of the depressive model group were not significantly changed compared to the healthy group. However, the expression of TPH1 mRNA was expressed at significantly lower levels in the liver and kidney tissues of the treated group than in the healthy group and depressive model group. The level of the TPH2 mRNA in the liver and kidneys tissue was not significantly changed in these experimental groups (Figure 4A, 4B). Based on these results, stress predominantly inhibited TPH1/2 expression in the brain.

### Stress promoted methylation of the *TPH1/2* gene in rat brains

**Figure 5:**
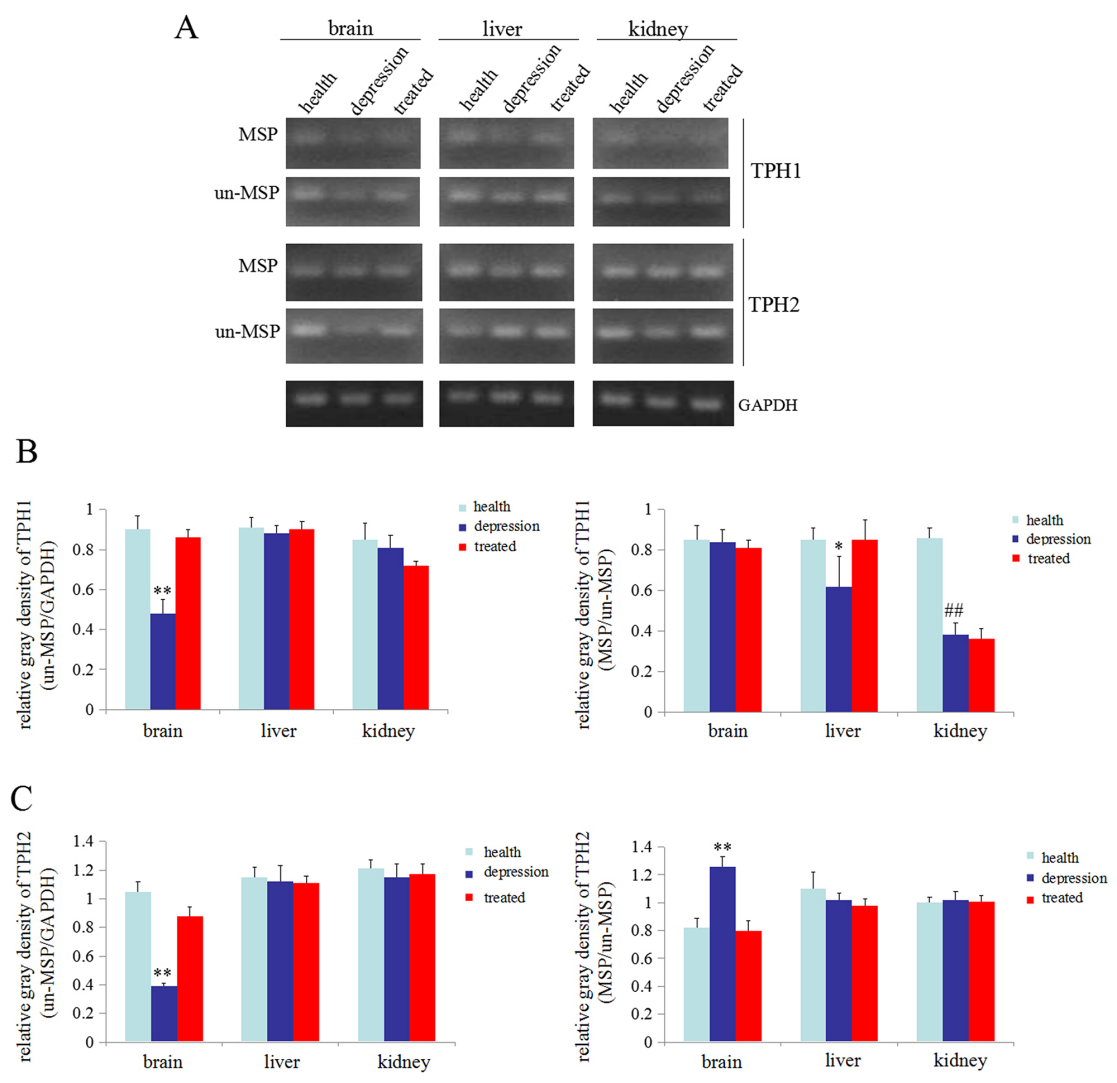
Methylation status of the *TPH1/2* genes in rats **(A)** Methylation-specific polymerase chain reaction (MSP) was used to assess the methylation status of the *TPH1/2* genes in the brain, liver, and kidney of healthy, depressive model and treated rats; GAPDH was used as an internal control. Relativequantity of TPH1 (from the images shown in A) **(B)** and TPH2 (from the images shown in A) **(C)** Un-MSP: un-methylated-specificPCR, MSP: methylated-specificPCR. **P<0.01 vs healthy and treated groups, ^##^*P*<0.01 vs healthy groups. The images are representative of six independent experiments.

In the present study, we used the un-MSP and MSP methods to analyze the promoters of the *TPH1/2* genes and determine whether stress inhibited the expression of the *TPH1/2* genes. One CpG island of the*TPH1* gene was identified upstream of bases −177 to −31 with a size of 147 bp, and one CpG island of the *TPH2* gene was identified upstream of bases −1983 to −1861 with a size of 123 bp. Based on the results from the un-MSP assay the *TPH1/2* genes were expressed at significantly lower levels in the brains of the depressive model group than in the healthy or treated group. The results of the MSP-PCR assay, indicated that the *TPH2* gene in the brain of the depressive model group was methylated to a significantly higher than in the healthy or treated group(MSP/un-MSP) (Figure 5A-5C). Unexpectedly, the level of methylation of the *TPH1* gene was significantly reduced in the liver of the depressive model group compared with the healthy or treated group, but in kidney tissues, the level of methylation of the*TPH1* gene was significantly reduced in the depressive model and treated groups than in the healthy group (Figure 5A, 5B). However, the methylation status of the *TPH2* gene was not altered in the liver and kidney tissues from the experimental groups (Figure 5A, 5C). Thus, stress inhibited the expression of the *TPH2* gene, possibly by promoting the methylation of these genes in the brain.

## DISCUSSION

Decreased expression of 5-HT in the central nervous system has been reported in patients with major depressive disorders, affective disorders, anxiety disorders, and other mood disorders [27, 28]. The concentration of 5-HT is associated with the development of depression disorder, and many reports have shown that tissue or serum 5-HT levels are low in patients with depression [29–31]. Inhibition of 5-HT reuptake also plays an important role in promoting depressive disorders [32]. Thus, the synthesis, transportation, and reuptake of 5-HT are pivotal factors that influence depressive-like behaviours. In the present study, we found that the tissue 5-HT concentrations were significantly decreased in the brain and liver tissues from rats in the stress-induced depressive model than in healthy rats, indicating the successful establishment of the depressive model. The 5-HT levels decreased in depression group and increased in treated rats. However, neither TPH1 nor TPH2 were correlated with the changes observed in the liver and kidney. We think the stress factors may primarily influence the expression of the TPH1 and TPH2 genes in brain tissues, TPH1 and TPH2 in the brain effect 5-HT biosynthesis, 5-HT accumulates in the liver and kidney tissues through the blood circulation. Paroxetine (Paxil) is an antidepressant drug that is used to treat major depressive disorder. The underlying mechanism of paroxetine has recently been shown to involve the inhibition of the activity of the 5-HT transporter, leading to the inhibition of 5-HT reuptake [33, 34], but researchers have not clearly determined whether paroxetine influences the expression of the rate-limiting enzymes in 5-HT synthesis, TPH1/2.

In the present study, stress could significantly inhibit TPH1/2 expression in the brain tissues of rats in the depressive model group in the immunohistochemistry assay, and the paroxetine treatment rescued TPH1/2 expression. We also found that TPH1/2 was expressed in the cytoplasm (Figure 2). Based on these results, paroxetine stimulates the expression of *TPH1/2* genes. Unexpectedly, stress did not inhibit the expression of the *TPH1/2* genes in the liver or kidney tissues, but after treatment with paroxetine the inhibition of stress in the expression of *TPH2* genes in brain were restored. Paroxetine is an antidepressant in a group of drugs called selective serotonin reuptake inhibitors (SSRIs), SSRIs block serotonin reuptake and increase serotonin stimulation of somatodendritic 5-HT1A and terminal autoreceptors. Because 5-HT biosynthesis is limited by TPH1/2, we explored the effect of paroxetine on TPH1/2 expression in liver and kidney tissues in the present study. After treatment with paroxetine, the TPH1 expression was significantly reduced in the liver and kidney tissues of the treated group than in the healthy or depressive model groups. Paroxetine did not affect TPH2 expression in these tissues (Figure 3). Based on these results, paroxetine selectively regulates TPH1/2 expression in different tissues, indicating a tissue-specific function of paroxetine. This study identified a novel mechanism by which paroxetine treats depressive disorder.

Epigenetic modulation of genes, such as DNA methylation and histone acetylation, affects the transcription of genetic information [35]. An increase in methylation within the promoter of a gene or an increase in histone acetylation leads to gene silencing in most cases [35–37]. We performed RT-PCR and MSP-PCR to detect the expression of the *TPH1/2* genes and the methylation status of *TPH1/2* gene promoters and to explore the mechanism by which stress inhibited the expression of the *TPH1/2* genes. The results showed that the expression of *TPH1/2* gene mRNAs in the brain tissues of the depressive model group was significantly decreased compared with the healthy or treated group. No changes in the expression of the *TPH1* mRNA were detected in the liver and kidney tissues of the depressive model group compared to the healthy group, but treatment with paroxetine significantly reduced the expression of this mRNA in these tissues compared to the depressive model group and the healthy group. The expression of the *TPH2* mRNA was not changed in the liver and kidney tissues of these experimental groups (Figure 4). Thus, stress predominantly inhibited *TPH1/2* mRNA expression in brain tissues. The MSP results also indicated that the *TPH2* gene promoter was methylated to a significantly greater extent in brain tissues from the depressive model group compared to the healthy or treated group. The methylation level of the *TPH1* gene promoter was significantly lower in the liver tissue from the depressive model than in the healthy or treated group. However, in kidney tissues, the depressive model group showed no changes compared to the treated group, although methylation was significantly lower in these groups than the healthy group. The methylation status of the *TPH2* gene promoter was not changed in liver and kidney tissues from these experimental groups (Figure 5). According to the results of the MSP assay, the decrease in TPH2 expression in the rats in the depressive model group may due to an increase in the methylation of the *TPH2* gene in brain tissues, consistent with the general function of DNA methylation. To our surprise, the MSP results indicated that the higher levels of methylation of TPH2 gene promoter in rats in the depressive model group, and THP2 expression negatively correlated, but a negative correlation between TPH1 methylation and expression was not observed. The reason may involve the specific expression of TPH1/2 in these tissues, and the observation that stress induced the differential methylation of the *TPH2* gene promoter in brain, liver and kidney tissues. We speculate that stress primarily induced changes in the methylation of the *TPH2* gene promoter in brain tissue, but mainly induced changes in the methylation of the *TPH1* gene promoter in liver and kidney tissues. Moreover paroxetine promoted *TPH2* expression by inhibiting the methylation of the *TPH2* gene promoter in brain tissues, leading to increase 5-HT synthesis.

## MATERIALS AND METHODS

### Animals and generation of the depressive model

For each group,thirty Sprague Dawley rats (180-220 g) with an equal number of males and females were used in our research. All newly acquired rats were firstly housed in 10 cages by sex. The control (group 1, 30 rats), depressive model (group 2, 30 rats), and treatment (group 3, 30 rats) groups were administered adaptive feeding for 7 days. Rats in group 1 were fed regularly, whereas rats in group 2 and 3 were were successively exposed to a variety of mild stressors, including tail suspension (1 min), stroboscopic light (120 shoots/min), noise (4 h), swimming in ice water (1 h) or hot water (45°C, 1 h), electrical stimulation (10 Hz), immobilization, food and water deprivation (24 h), for three days each for 15 days. Subsequently, groups 2 and 3 were treated (gavaged) with saline and Paxil (paroxetine) (Zhejing Jianfeng Pharmaceutical Factory, Jinhua, Zhejiang Province, China) at 0.2 mg/ml/day, respectively, for 30 days. After anesthetization and dissection, the brain, liver and kidney of each rat were separated and stored at −80°C until further experiments.

### Analysis of 5-HT concentrations using an enzyme-linked immunosorbent assay (ELISA)

Concentrations of 5-HT in the whole brain, liver, and kidney tissues of healthy, depressive, and treated rats were determined using enzyme-linked immunosorbent assay(ELISA) with a Covalent Binding Surfaces Plate (Thermo Scientific, 436006). Four repeats were established for both total protein extracted from the tissues and standards. The standard curve was generated by measuring the absorbance of 5-HT (Sigma-Aldrich, 14927) solutions with final concentrations of 31.25 ng, 62.5 ng, 125 ng, 250 ng, 500 ng, and 1000 ng. Coating buffer (Sangon, E661004-0010) was used as a diluent to immobilize the protein. Protein coating was performed at 4°C overnight following three washs with phosphate buffer (PBS) for 5 min each (5 min×3). Proteins were then blocked with skim milk for 1 h at 37°C following three washs by PBST(PBS with Tween-20) for 5 min each. The plates were washed with PBST three times for 5 min each and then the primary anti-5-HT antibody (Abcam, AB6444) was added at a dilution of 1:1000, and incubated at 37°C for 1 h. After 3 washes with PBST for 5 min each, the secondary antibody (goat anti-rabbit) was added at a 1:1000 dilution and incubated at 37°C for 1 h. Finally, visualization was performe dusing 1-step Ultra TMB-ELISA Substrate Solution (Pierce, 34028). The absorbance of each well was measured at 450 nm by an iMark Microplate Reader (Bio-Rad, 15594).

### Immunohistochemistry analysis of TPH1/2 expression

Immunohistochemistry was performed to visualize and compare the distributions of TPH1/2 in healthy, depressive and treated rats. Anti-TPH1 (Thermo Fisher Scientific, PA1-777) and anti-TPH2 (Abcam, AB184505) antibodies were used in these three sets of experiments, to detect the target proteins in paraffin sections of the brain, liver and kidney. The thickness of each section was 4 μm to obtain a clear view of the intact tissue. All sections were mounted on gelatinized glass slides and treated identically. The sections were initially deparaffinized with 3 rinses in xylene for 5 min each, and rehydrated with a descending ethanol gradient (100%, 95% and 70%) for 3 min each. After 5 min×2 washes with distilled water, all sections were incubated in endogenous peroxidase blocking solution (3% hydrogen peroxide) for 5 min at room temperature and rinsed with running water for 5 min. Subsequently, the sections were blocked by incubating them with 2% bovine serum albumin (BSA) for 10 min and washed 3 times with PBS for 10 min each. Antigen retrieval was then performed in citrate buffer by microwaving the sections 6 times for 10 s each. After another wash with running water for 5 min, the sections were incubated overnight at 4°C with the antibodies against TPH1 diluted 1:140 in PBS, and antibodies against TPH2 diluted 1:250 in PBS. The reaction was terminated by washing the sections 3 times with PBST for 10 min each. The signals from bound primary antibodies were amplified by incubating the sections with secondary antibodies (goat anti-rabbit for TPH1/2, both diluted 1:500) for 30 min at 37°C. The reaction was terminated by washing the sections 3 times with PBST for 10 min each, the peroxidase label was visualized by incubating the sections with 3,3′-diaminobenzidine (DAB) solution (Solarbio, DA1010) for 2-10 min at room temperature. Paraffin sections were counter-stained with haematoxylin (KeyGEN, KGA223) for 3 min at room temperature following a rinse with running water for 5 min. Finally, the specimens were washed with running water, dehydrated with an increasing gradient of ethanol, cleared in xylene, and mounted with neural balsam. Photographs of all stained sections were captured with a microscope (Olympus, CKX41).

### Western blotting analysis of TPH1/2 expression

Western blotting was performed to detect the distributions of the TPH isoforms and to semi-quantitatively analyze the levels of the target proteins in the healthy, depressive model and treated rats. Proteins were extracted using a Whole Cell Lysis Assay Kit (KeyGEN, KGP2100), and protein concentration was then measured with a Bradford assay. Fifty micrograms of total protein were loaded in each lane. After transferring the proteins to a polyvinylidene fluoride (PVDF) membrane with a voltage of 40 V for 3 h at 4°C, the membrane was blocked with 5% skim milk at room temperature for 2 h. The membrane was then incubated with primary antibodies against TPH1 (Thermo Fisher Scientific, PA1-777) and TPH2 (Abcam, AB52954) at a final dilution of 1:500 overnight at 4°C. On the second day, the membrane was washed and incubated with the goat anti-rabbit antibody at room temperature for 2 h. Visualization was performed by adding ECL Western Blotting Substrate (Thermo Pierce, 32209), and bands were observed on a fluorescent/chemiluminescent detector (Tanon, 4500).

### Analysis of the TPH1/2 mRNA levels using reverse transcription-polymerase chain reaction (RT-PCR)

RT-PCR was performed to determine whether the TPH mRNA levels were directly proportional to TPH1/2 expression in the brains of the healthy,depressive model and treated rats. Total RNA was extracted from the brains using an RNeasy Plus Mini Kit (Qiagen, 74134). Reverse transcription was performed with a PrimeScript RT-PCR Kit (TaKaRa, DRR014A) using random 6 mers (random 6-base primer for the transcription of DNA into rRNA, mRNA or tRNA) as primers. The mRNA was then amplified with Recombinant Taq DNA Polymerase (TaKaRa, R001AM) using gene-specific primers for TPH1, TPH2 and 18S ribosomal RNA (18s rRNA; used as an internal reference and positive control for RT-PCR). The primer sequences and size of the target sequences for RT-PCR are listed in Table 1. TPH1 and TPH2 primers were designed using the Primer Premier 5 software and were designed to span across exons and introns to exclude potential DNA contamination. The PCR was initiated at 95°C for 5 min, and the amplifications were performed with 38 cycles at 95°C for 30 s (denaturation), 60°C for 30 s (annealing), and 72°C for 1 min (extension), followed by an extra extension step at 72°C for 10 min. The PCR products were then visualized with 1% agarose gels stained by Gold View (Solarbio, G8142) and viewed in a fluorescent/chemiluminescent detector (Tanon, 4500).

**Table 1:** Primers for RT-PCR

mRNA (accession number)	Primer sequence	Product length (bp)
18s rRNA	5′-CCTGCGGCTTAATTTGACTC-3′ (sense)	118
(M11188)	5′-AACTAAGAACGGCCATGCAC-3′ (antisense)	
TPH1	5′-GCTGAACAAACTCTACCCAACCCAC-3′ (sense)	427
(NM_001100634)	5′-TCGGCACAGTCCAAACTCCACA-3′ (antisense)	
TPH2	5′-AATCTTCGTGGACTGTGAATGTG-3′ (sense)	449
(NM_173839)	5′-CGTTGTCTTCCCTGTAGCCG-3′ (antisense)	

### Methylation-specific polymerase chain reaction (MSP) analysis of methylation status of the *TPH1/2* genes

Methylation-specific PCR (MSP) was performed to investigate the methylation of the *TPH1/2* gene promoters within the CpG islands located at the 5′-terminal, which may inhibit the transcription of *TPH1/2* in the healthy, depressive model and treated rats. Methylation-specific primers and non-methylation specific primers were designed by Sangon Biotech Company. Total DNA was extracted from the whole brain, liver and kidney of the healthy, depressive model and treated rats using a DNeasy Blood & Tissue Kit (Qiagen, 69504), and the final elution was performed by adding 100 μl of DNase-free water. A TIANquick Midi Purification Kit (Tiangen, DP204) was used to further purify the DNA. Finally, 30 μl of the purified DNA product was obtained. DNA was sulfurated with a CpGenome Turbo Bisulfite Modification Kit (Merck, S7847). DNA was then amplified with Recombinant Taq DNA Polymerase (TaKaRa, R001AM) using methylation-specific primers and non-methylation specific primers. The primer sequences and size of the target sequences for PCR are listed in Table 2. PCR was initiated at 95°C for 5 min, and amplifications were performed by 33 cycles at 95°C for 30 s (denaturation), 52.5°C for 30 s (annealing), and 72°C for 1 min (extension), followed by an extra extension at 72°C for 10 min. The PCR products were then visualized with 1% agarose gels stained by Gold View (Solarbio, G8142) and semi-quantitatively analyzed using a fluorescent/chemiluminescent detector (Tanon, 4500).

**Table 2:** Primers for MSP

Gene		Primer sequence	Product length (bp)
*TPH1*	Methylation- specific primers	5′-AATTAAAATTTTACGAGAATAGTTGTG-3′ (sense) 5′-AACGCAAAAAAAAACAACTAATAA -3′ (antisense)	154
	Non-methylation- specific primers	5′-TAATTAAAATTTTATGAGAATAGTTGTG -3′ (sense) 5′-CACAAAAAAAAACAACTAATAAAAA -3′ (antisense)	153
*TPH2*	Methylation- specific primers	5′-GTGTTTGTAGTATTGATTATTTCGTG -3′ (sense) 5′-TAAAAAAATCAAATTATACGATAACC -3′ (antisense)	103
	Non-methylation- specific primers	5′-AGTGTTTGTAGTATTGATTATTTTGTG -3′ (sense) 5′-ACTAAAAAAATCAAATTATACAATAACC -3′ (antisense)	106
*GAPDH*		5′-GACATGCCGCCTGGAGAAAC-3′ (sense) 5′-AGCCCAGGATGCCCTTTAGT-3′ (antisense)	92

### Statistical analysis

Data are presented as mean ± S.D. The statistical analysis was performed using Student's *t* test (for two experimental groups). Significance was set at *P*<0.05. Statistical significance was determined using Student’ s*t* test and the *F* test (SPSS 11.5 software for Windows, SPSS Inc., Chicago, IL, USA).

## CONCLUSIONS

As shown in the present study, 1) the two isoforms of TPH have a different spatial distribution, with TPH1 expressed predominantly in the peripheral tissues and TPH2 primarily localized in the central serotonergic neurons; 2) Tissues 5-HT concentrations and TPH1/2 expression were decreased in brain tissues in the rat model of depression; 3) Stress inhibited TPH2 expression in the depressive rats possibly due to an increase in the methylation of the *TPH2* gene promoter in brain tissues. Our results confirmed that 5-HT and TPH1/2 dysfunction were associated with depression. Notably, the stress-mediated decrease in TPH1/2 expression was related to increased methylation of the *TPH1/2* gene promoters.
